# *Tapirira guianensis* Aubl. Extracts Inhibit Proliferation and Migration of Oral Cancer Cells Lines

**DOI:** 10.3390/ijms17111839

**Published:** 2016-11-08

**Authors:** Renato José Silva-Oliveira, Gabriela Francine Lopes, Luiz Fernando Camargos, Ana Maciel Ribeiro, Fábio Vieira dos Santos, Richele Priscila Severino, Vanessa Gisele Pasqualotto Severino, Ana Paula Terezan, Ralph Gruppi Thomé, Hélio Batista dos Santos, Rui Manuel Reis, Rosy Iara Maciel de Azambuja Ribeiro

**Affiliations:** 1 Molecular Oncology Research Center, Barretos Cancer Hospital, Barretos 14784-400, Brazil; renatokjso@gmail.com (R.J.S.-O.); ruireis.hcb@gmail.com (R.M.R.); 2 Laboratory of Experimental Pathology, Federal University of São João del Rei—CCO/UFSJ, Divinópolis 35501-296, Brazil; gabrielafrancine_lp@hotmail.com; 3 Laboratory of Mutagenesis, Federal University of São João del Rei—CCO/UFSJ, Divinópolis 35501-296, Brazil; luizfcamargos@yahoo.com.br (L.F.C.); fabiosantos@ufsj.edu.br (F.V.d.S.); 4 Medical School, Federal University of Minas Gerais—UFMG, Belo Horizonte 31270-901, Brazil; anamacielr@gmail.com; 5 Special Academic Unit of Physics and Chemistry, Federal University of Goiás, Catalão 75704-020, Brazil; richeleps@ufg.br (R.P.S.); vanessa.pasqualotto@gmail.com (V.G.P.S.); apterezan@hotmail.com (A.P.T.); 6 Laboratory of Tissue Processing, Federal University of São João del Rei—CCO/UFSJ, Divinópolis 35501-296, Brazil; ralphthome@gmail.com (R.G.T.); hbsufsj@gmail.com (H.B.d.S.); 7 Life and Health Sciences Research Institute (ICVS), Health Sciences School, University of Minho, Braga 4710-057, Portugal; 8 3ICVS/3B’s-PT Government Associate Laboratory, Braga 4710-057, Portugal

**Keywords:** HNSCC, cytotoxic activity, alkaloids, apoptosis markers

## Abstract

Cancer of the head and neck is a group of upper aerodigestive tract neoplasms in which aggressive treatments may cause harmful side effects to the patient. In the last decade, investigations on natural compounds have been particularly successful in the field of anticancer drug research. Our aim is to evaluate the antitumor effect of *Tapirira guianensis* Aubl. extracts on a panel of head and neck squamous cell carcinoma (HNSCC) cell lines. Analysis of secondary metabolites classes in fractions of *T. guianensis* was performed using Nuclear Magnetic Resonance (NMR). Mutagenicity effect was evaluated by Ames mutagenicity assay. The cytotoxic effect, and migration and invasion inhibition were measured. Additionally, the expression level of apoptosis-related molecules (PARP, Caspases 3, and Fas) and MMP-2 was detected using Western blot. Heterogeneous cytotoxicity response was observed for all fractions, which showed migration inhibition, reduced matrix degradation, and decreased cell invasion ability. Expression levels of MMP-2 decreased in all fractions, and particularly in the hexane fraction. Furthermore, overexpression of FAS and caspase-3, and increase of cleaved PARP indicates possible apoptosis extrinsic pathway activation. Antiproliferative activity of *T. guianensis* extract in HNSCC cells lines suggests the possibility of developing an anticancer agent or an additive with synergic activities associated with conventional anticancer therapy.

## 1. Introduction

Cancer of the head and neck is a group of upper aerodigestive system neoplasms that corresponds to the seventh most common cancer worldwide [1]. They are aggressive tumors and can often be invasive and metastatic [2]. This pathology is a condition that continues to be diagnosed in an advanced stage with low survival rate [3].

The prevalence of oral cancer in Brazil is high, especially among men in their fifth decade of life [4]. According to INCA (Brazilian National Cancer Institute), approximately 300,000 new cases of oral cancer were diagnosed in 2012 and it was responsible for 145,000 deaths. The five-year survival rate of these patients is 50%–60% [4,5,6]. Unfortunately, even aggressive treatments, such as surgery, radiotherapy and chemotherapy are not curative, and cause severe co-morbidities. To overcome these limitations, the phytomedicine, which uses therapeutic agents derived from plants as an adjuvant treatment in combination with surgery and or radiotherapy, can constitute potential options [7,8].

Some plants are known to contribute significantly within the traditional medicine of developing countries [9,10]. In the last decade, research on natural compounds has been particularly successful in the field of anticancer drug research. Examples of anticancer agents developed from higher plants (also known as vascular plants) are the alkaloids vinblastine and vincristine, which were both obtained from the Madagascar periwinkle (*Catharanthus roseus*) [11,12]. Brazil is home of the greatest biodiversity, representing more than 20% of the total number of species on Earth [13]. This fact, together with the need for developing new drugs, justifies the incentive for research programs in investigation of potential medicinal plants.

*Tapirira guianensis* Aubl., a member of the Anacardiaceae family, is popularly known in Brazil as “tatapiririca”, “cedroí” or “Pau-pombo”. It is widely distributed throughout the Brazilian territory, and its leaves and bark are used by folk medicine to treat dermatitis, syphilis and as a cleanser, due to its antibacterial and antifungal activity [14]. Preliminary study demonstrated that the CHCl_3_ extract and two isolated compounds of the seeds of *T. guianensis* displayed cytotoxicity activity against human prostate cancer [15], however the cytotoxic action of the leaves constituents remain unknown.

Studies have shown the presence of secondary compounds of the families of tannins, coumarins, flavones, flavonols, flavanones, saponins, steroids and alkaloids in *T. guianensis* leaves [16,17]. The presence of hidrobenzofuranides norisoprenoids that are long chain metabolites with suggested anti-tumor activity was also identified in the leaves [17]. Longatti et al. found that crude extract and the hydroalcohol, chloroform and ethyl acetate fractions showed significant inhibition of matrix metalloproteinase 2 (MMP-2) [16].

Despite these findings, the knowledge of *T. guianensis* anti-neoplastic activity and its molecular mechanisms is still scarce. Therefore, in this study, we aim to evaluate the effect of this species in head and neck tumor cell lines.

## 2. Results

### 2.1. Nuclear Magnetic Resonance (NMR) Analysis of T. guianensis

The identification by ^1^H NMR of secondary metabolites classes present on the crude extract and fractions obtained by liquid–liquid extraction from *T. guianensis* was based on their chemical shifts (ppm) features.

The NMR spectrum of the crude extract (C3) presented signals of chemical shifts characteristics of alkaloids, coumarins and flavonoids in aromatic region of 6.0–7.5 ppm, hydrogen bonded to carbon sp^2^ and/or neighbors heteroatoms in region of 3.4–5.3 ppm for alkaloids and coumarins and sugars (saponin and/or glycosylated flavonoids) and hydrogen bonded to carbon sp^3^ in region of 0.7–2.4 ppm for saponin and steroids. The ethyl acetate fraction (C1) showed signals of chemical shifts typical of coumarins and flavonoids in aromatic region of 6.0–7.3 ppm, hydrogen bonded to carbon sp^2^ and/or neighbors heteroatoms in region of 3.0–5.8 ppm for coumarins and glycosylated flavonoids, and hydrogen bonded to carbon sp^3^ in region of 0.5–1.5 ppm for steroids. The hydroalcoholic fraction (C2), on the other hand, exhibited signals of chemical shifts characteristics of alkaloids, coumarins and flavonoids in aromatic region of 6.2–8.2 ppm, hydrogen bonded to carbon sp^2^ and/or neighbors heteroatoms in region of 3.2–5.4 ppm for alkaloids, coumarins and sugars (saponin and/or glycosylated flavonoids) and hydrogen bonded to carbon sp^3^ in region of 0.8–2.3 ppm for saponin and steroids. Lastly, the hexane fraction (C4) displayed signals of chemical shifts typical of alkaloids in aromatic region of 6.0–7.3 ppm and hydrogen bonded to carbon sp^2^ and/or neighbors heteroatoms in region of 3.0–5.8 ppm, a hydrogen bonded to carbon sp^3^ in region of 0.5–1.5 ppm for steroids.

### 2.2. Ames Mutagenicity Assay

All evaluated doses of *T. guaianensis* hydroalcoholic extract induced a significant increase in revertants frequency employing the TA98 strain of *Salmonella typhimurium* (Table 1). The Ames test mutagenic index (MI) values observed ranged between 3.37 (to lowest dose) and 14.84 (to highest dose), indicating a high mutation index of this extract. However, in the TA100 strain without S9, the mutagenicity was not observed. None of the assessed doses induced significant alterations in revertants frequency to this strain when compared with negative control group dimethyl sulfoxide (DMSO) and all MI values were lower than 2. Nevertheless, in studies with TA100 with metabolic activation (+S9), the analyzed extract was mutagenic, but at a lower level, suggesting the presence of indirect mutagens that induce base substitutions in DNA structure.

### 2.3. Efficacy of T. guianensis Samples in Head and Neck Squamous Cell Carcinoma Cell Lines

The half-maximal inhibitory concentration (IC_50_) was determined to assess the cytotoxicity of *T. guianensis* samples in head and neck tumor cell lines (Table 2) and a representation of the proliferation and survival curves of the head and neck tumor cell lines is depicted in Figure S1. The ethyl acetate fraction (C1) showed IC_50_ values between 28.0 ± 2.9 and 244.0 ± 4.8 µg/mL. The hydroalcoholic fraction (C2) exhibited low values 14.0 ± 2.0 and 15.0 ± 2 µg/mL for SCC14 and HN13 cell lines, respectively. The crude extract of *T. guianensis* (C3) display IC_50_ values between 45.0 ± 4.0 and 349.0 ± 5.6 µg/mL. The hexane fraction (C4) showed an IC_50_ values varying from 58.0 ± 1.9 to 592.0 ± 20 µg/mL. We observed that the SCC25 cell lines exhibited a resistant phenotype for all compounds with IC_50_ values varying from 240 ± 26.0 to 592.0 ± 2.0 µg/mL (Table 2).

**Figure 1:**
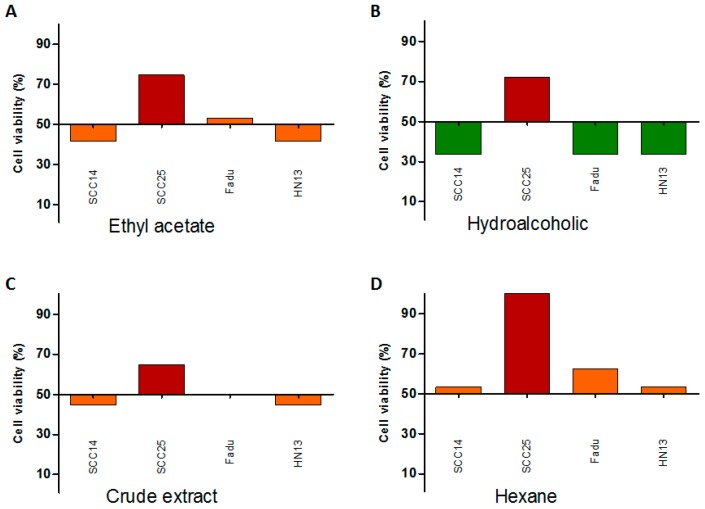
Cytotoxicity profile of SCC14, SCC25, Fadu, HN13 head and neck tumor cell lines, exposed to the different fractions of the *T. guianensis* extract: (**A**) Ethyl acetate (C1); (**B**) Hydroalcoholic (C2); (**C**) Crude extract (C3); and (**D**) Hexane (C4). Bars represent the cell viability at 50 µg/mL. Bars represent the GI score classification. Green (HS: highly sensitive); Orange (MS: moderate sensitive) and Red (R: resistant).

We further evaluated the growth inhibition (GI) classification and showed a moderate sensitivity (MS) and highly sensitive (HS) for ethyl acetate (C1) and hydroalcoholic fractions (C2) in SCC14, Fadu and HN13 cell lines, respectively (Table 3). The crude extract (C3) and hexane fraction (C4) showed a moderate sensitivity in SCC14, Fadu and HN13 cell lines (Table 3). As expected, the SCC25 cell line displayed a resistant (R) phenotype for all compounds (Table 3; Figure 1). Based on these results, the most sensitive cell line (HN13) was chosen for the subsequent cellular characterization.

### 2.4. Wound Healing Migration Assay

**Figure 2:**
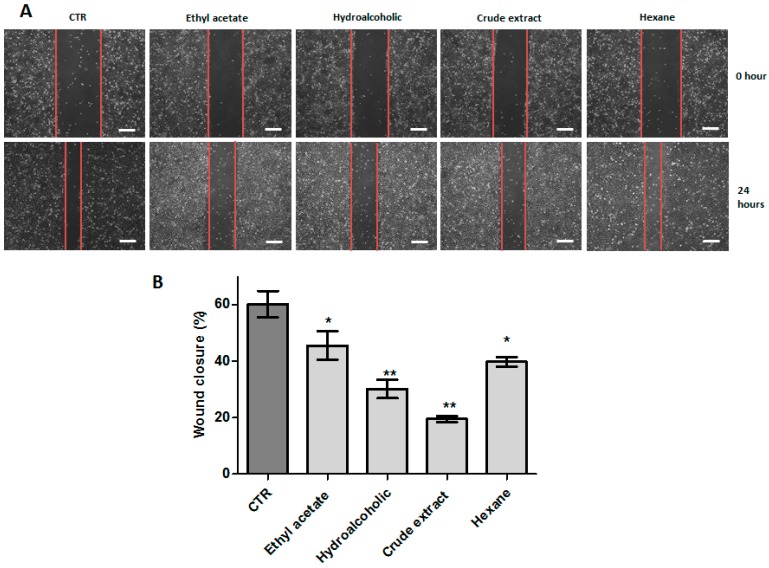
(**A**) Representative images of wound healing assay of HN13 cell line after 24 h, exposed to ethyl acetate fraction (25 μg/mL), hydroalcoholic fraction (10 μg/mL), crude extract (50 μg/mL) and hexane fraction (60 μg/mL). The red lines represent the distance between both edges of the wound; Scale bars, 200 μm; (**B**) bars represent the relative migration expressed as the means ± SD for different compounds. All experiments were repeated for three times. * *p* < 0.05; ** *p* < 0.01.

In the HN13 cell line, we observed that after 24 h, 60.20% ± 9.35% of the control (DMSO) wound was closed (Figure 2A,B). All fractions and extract exhibited a significant reduction of wound closure, with the hydroalcoholic (C2) and crude extract (C3), showing the highest closure rate, namely 30.21% ± 5.81% and 19.54% ± 2.03%, respectively (*p* < 0.01) (Figure 2A,B).

### 2.5. Invasion Assay

**Figure 3:**
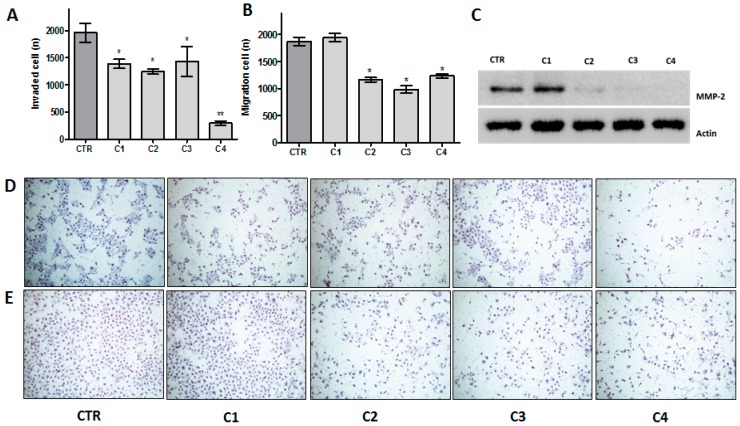
Effect of crude extract (C3) and fractions (C1, C2 and C4) of *T. guianensis* on HN13 cellular migration and invasion. (**A**) Invasion was measured at 24 h by Matrigel invasion assay, the results were expressed in relation to the DMSO control (considered as 100% of invasion) as the mean percentage of invasion ± SD; (**B**) analysis of index cell migration measured at 24 h by trans-well, the results are expressed as the means ± SD; (**C**) western blot analysis using the antibodies of anti-human MMP-2. The cells lines were incubated with compounds for 24 h; (**D**,**E**) representative images (at ×40 magnification) of migration and invasion assays, respectively. * *p* < 0.05; ** *p* < 0.01.

We further addressed whether *T. guianensis* extract and fractions could impair cell migration and invasion in vitro (Figure 3). Using matrigel invasion assay, all compounds reduced the invasion of HN13 cell lines, however the C4 fraction showed greater effect on cell invasiveness ability by matrix degradation (*p* < 0.01) (Figure 3A,D). We previously observed the migration inhibition induced by the hydroalcoholic (C2) and crude extract (C3) by wound healing assay. Additionally, we assess migration potential using empty inserts (without Matrigel), where we found a significant reduction of the migratory potential exposed to the hydroalcoholic (C2), crude extract (C3) and hexane (C4) fractions. Finally, no significant differences were detected between C2 and C4 (Figure 3B,E). In order to interrogate whether the invasion capability was associated with metalloproteinase activity, we analyzed MMP-2 protein expression by Western blot that showed the presence of the MMP-2 (72 kDa) band only in the C1 treatment (Figure 3C).

### 2.6. Expression of Apoptosis Markers (PARP, Caspase 3, and Fas)

**Figure 4:**
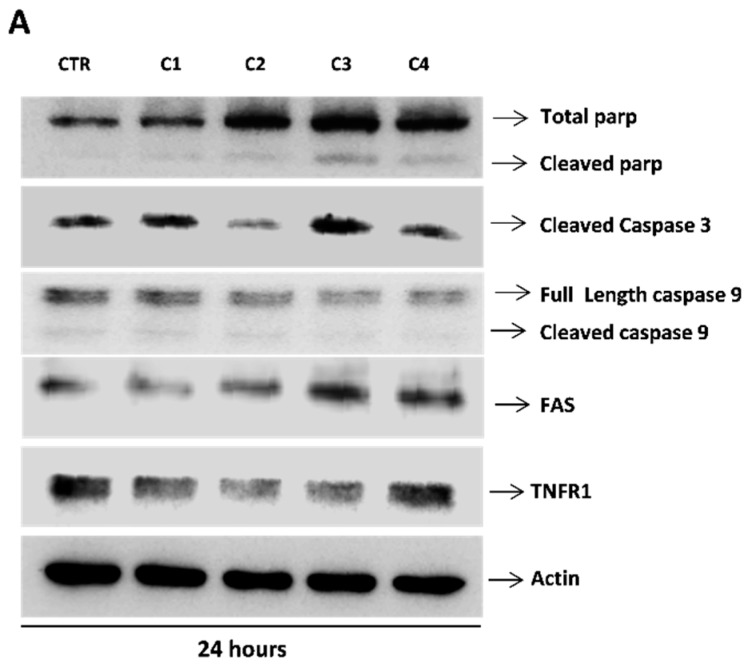

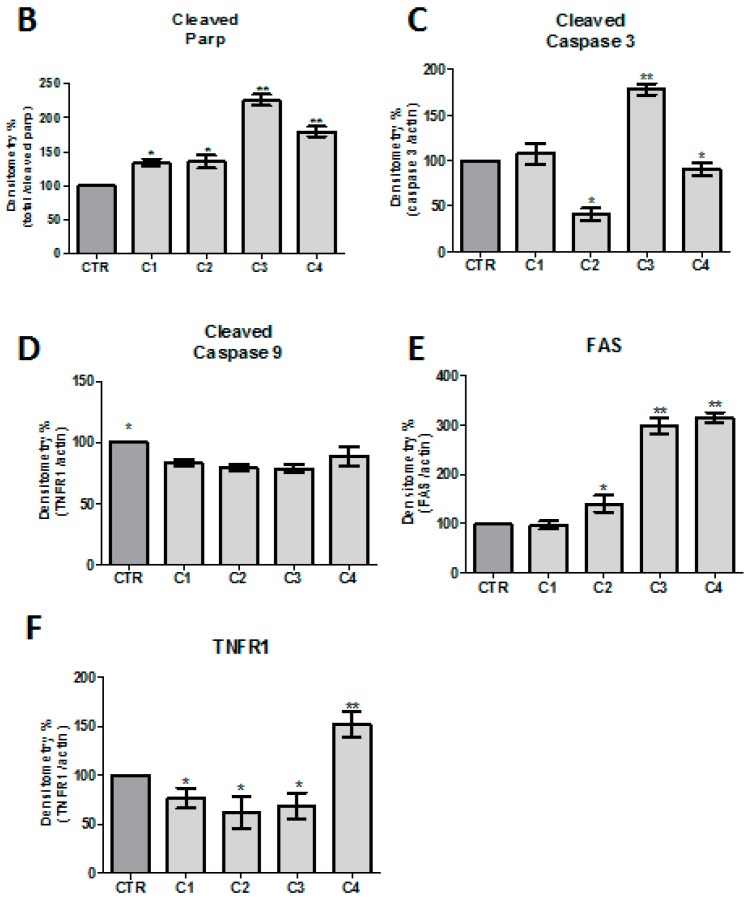
Effect of crude extract (C3) and fractions (C1, C2 and C4) of *T. guianensis* on intracellular signaling pathway activation in HN13 cells. HN13 cell lines were incubated with compounds (IC_50_ values) for 24 h. (**A**) Western blot, using the antibodies of anti-human PARP (Full Length: 116 kDa and cleaved: 89 kDa), Caspase 3 (Full Length: 35 kDa), Caspase 9 (cleaved: 19 kDa) Fas (40 kDa) and TNFR1 (55 kDa) and Actin (45 kDa); (**B**–**F**) densitometric analysis of Western blot data of the four proteins. Bars results are expressed as the means ± SD. * *p* < 0.05; ** *p* < 0.01.

To elucidate whether crude extract and fractions induce cancer cell death, HN13 cells were treated for 24 h, and the expression of total and cleaved PARP, caspase 3, FAS and tumor necrosis factor receptor 1 (TNFR1) proteins was evaluated by Western blot (Figure 4). All compounds showed a significant increase of cleaved PARP compare to control (DMSO), being more significant for the crude extract (C3) and hexane fraction (C4) (Figure 4B). Accordantly, we found increased levels of caspase 3 in HN13 cell lines treated with crude extract (C3) and hexane fraction (C4) (Figure 4C). Interestingly, we found significant increased levels of FAS in the C3 and C4 fraction (Figure 4D), and only the C4 fraction showed increasing levels of TNFR1 (Figure 4E).

## 3. Discussion

In the present study, we have reported the antineoplastic potential of the crude extract and fraction of *T. guianensis* that showed a significant cytotoxicity effect in a panel of HNSCC cell lines. These data were further supported by results from migration and invasion assay, which demonstrated a significant reduction in migration index and cellular invasion. Moreover, the analysis of key cell death players indicates an activation of the extrinsic apoptotic pathway.

A variety of studies conducted by the NCI (National Cancer Institute), showed that more than 1000 different phytochemicals have cancer-preventive activity [18] Brazil contains the richest flora worldwide, and the great majority of it is unexplored in terms of therapeutic proprieties.

We initiated this study by assessing the mutagenicity of *T. guianensis* crude extract, using the Ames assay. The hydroalcoholic extract was mutagenic in TA98 strain without and with metabolic activation. High values of mutagenic index were observed in these conditions, indicating that compounds present in the extract are able to induce gene mutations. Nevertheless, in TA100 strain of *S. typhimurium*, the extract was mutagenic only after metabolic activation (+S9). According to Mortelmans and Zeiger (2000), the strain TA98 identifies agents able to induce gene mutations by deletions or additions of nucleotides on DNA structure, resulting in frameshift mutations [19]. Besides, TA100 strain detects compounds that cause base substitution in DNA. Considering these findings, it was identified that the crude extract presents in its phytochemical constitution agents able to induce frameshift mutations directly or indirectly. However, studies performed in TA100 indicated that substitution of bases was induced by the extract only after its modification by hepatic enzymes, present in the S9 fraction.

Several studies have shown that some flavonoids can be potent mutagenic agents in different biological systems [20,21]. In accordance with Procházková et al. (2011), the antioxidant or pro-oxidant effects of flavonoids are related with the concentration during exposition and particular molecular characteristics [22]. The values indicated in these tests may suggest the mechanisms of action of the mutagenic activity evidenced in the extracts. However, the concentrations of growth inhibition of the different cell lines do not relate with mutagenic assay.

A previous study of seeds of *T. guianensis* isolated two cyclic compounds which were broadly active against a panel of human cancer cell lines [12]. Based on putative anti-tumoral effect of *T. guianensis*, we tested its anti-proliferative action in a panel of four head and neck cancer cell lines Briefly, we showed that all fractions caused a moderate (SCC14 and Fadu) or high (HN13) cytotoxicity, with exception of the SCC25 cell line, which showed a resistant profile. We observed the classification and response to different fractions from extract of *T. guianensis* in head and neck tumors cell lines and verified that hydroalcoholic fraction showed the best results with low IC_50_ values (14.0 ± 2.0 and 15.0 ± 2 µg/mL). The NMR analyses showed signals of chemical shifts characteristics of alkaloids, coumarins and flavonoids in hydroalcoholic fraction (C2). Flavonoid has been identified in crude extract of leaves of the *T. guianensis*. In our study, rutin and quercetin were the flavonoids identified, the latter present in greater quantities. These results are in agreement with previous report that detected these components [16]. Moreover, in vitro studies have found the anticancer effects of flavonoids in the NPC nasopharyngeal carcinoma cell line [23]; K562 leukemia cell line [24,25]; 184-B5/HER normal breast cell line [26] and MCF-7 breast cancer cell line [27]. Furthermore, in vivo approaches indicated that flavonoid compounds, similar to hydrobenzofuranoids obtained from *T. guianensis*, exhibit similar biological activity [26,27]. Therefore, we suggest that the presence of flavonoids compounds within the extract used in this study can be related to cytotoxic effects.

Cell motility is considered to be an important determinant of the metastatic potential of cancer cells and can constitute promising treatment strategy [26,28]. Therefore, we used different experiments to evaluate the effects of *T. guianensis* extract and fractions on the motility, migration and invasion. We detected significant results in wound closure of the HN13 cell line exposed to all compounds. Importantly, the hydroalcohol (C2), crude extract (C3) and hexane (C4) compounds, which showed high levels of flavonoids such as quercetin, showed reduced invasion on the transwell migration assay. Previous in vitro study showed that suppression of the HGF/c-Met signaling pathway by quercetin, contributes to the anti-metastatic action and inhibit the cellular migration in melanoma cell lines [29] and also inhibited the Wnt pathway in teratocarcinoma cell line [28].

We also showed that all compound inhibited the invasion in matrigel assay. MMPs are known ECM-degrading enzymes and theirs activity are associated with tumor invasiveness [30] and associated with tumorigenesis, progression and prognosis of head and neck tumor [31,32,33]. Herein, we observed that hydroalcohol (C2), crude extract (C3), and hexane (C4) fractions induced MMP-2 downregulation. These results are in line with previous work from our group that found that extracts of *T. guianensis* inhibited matrix metalloproteinases [16].

Our findings suggest that several classes of secondary metabolites of C2, C3 and especially C4 fraction may be responsible for the inhibition of cellular proliferation. Total alkaloids found in these compounds can be related with reduced invasion. A recent study showed that total alkaloids from *Rubus alceifolius* Poir inhibited the enzyme activity and reduced the expression of MMP-2 and MMP-9 [34]. Alkaloids are secondary compounds that exhibit anti-tumor activity with broad spectrum of action and they deserve further and deeper studies. Other studies have shown that alkaloids derived from other plants also present antitumor activity, such as opium alkalois (papaverine, noscapine and narceine) that induces apoptosis in cancer cell lines [35], capsaicin (an alkaloid isolated from the chili pepper) that modulates cell cycle progression and induces apoptosis in human KB cell line (epidermoid carcinoma) through mitochondrial membrane permeabilization and caspase activation, suggesting an antineoplastic activity [36]. We believe that our results offer important perspectives on cancer targeted therapy, especially in the control of metastasis mechanisms, exhibit in vitro for C2, C3 and C4 compounds.

Apoptosis pathway was assessed by Western blot, where we detected an overexpression of FAS, caspase-3, TNFR1 and increased level of cleaved PARP in C3 and C4, which indicates a possible extrinsic pathway of apoptosis. Fas-mediated apoptosis, ligands of Fas leads to strong caspase-8 activation at the DISC, thereby activating other caspases including caspase-3 in the absence of mitochondrial involvement [37]. Despite the absence of moderate anti-neoplastic effect of the C3 and C4 fraction, these fractions have secondary metabolites that activate an extrinsic activation mechanism mediated by FAS and TNFR1 increased. Nevertheless, the mechanisms in which *T. guianensis* participates into the complex signal pathways to achieve its anticancer role need further investigation, such as monitoring of other proteins involved in extrinsic apoptotic signaling

## 4. Materials and Methods

### 4.1. Plant Material and Extraction

Samples of the *T. guianensis* were collected within the Cerrado region of Minas Gerais, Brazil (Latitude S18°58′08″ and Longitude W49°27′54″). After identification, a voucher specimen was registered (143407) in the Herbarium of the Department of Botany, of the Federal University of Minas Gerais, in Belo Horizonte (Brazil).

Dried and powdered leaves (100 g) of *T. guianensis* were extracted by maceration (70% hydroalcoholic solution, 15 days), which was filtered and afterwards lyophilized, resulting in 2.111 g of crude extract. The extract was dissolved in CH_3_CH_2_OH/H_2_O (7:3) and successively extracted with hexane (C_6_H_14_), chloroform (CHCl_3_) and ethyl acetate (C_4_H_8_O_2_), resulting in 1.1059 g (4677%), 0.3904 g (20.04%) and 0.2321 g (11.92%), respectively, and the hydroalcoholic residue was 0.2363 g (12.13%) [12]. We further used the fractions: ethyl acetate (C1), hidroalcoholic (C2), crude extract (C3) and hexane (C4).

### 4.2. Nuclear Magnetic Resonance (NMR) Analysis of T. guianensis

The extract and fractions from *T. guianensis* were prepared with deutered solvents for nuclear magnetic resonance (NMR) analysis. The ^1^H NMR spectra were obtained using Bruker DRX-400MHz spectrometer (Bruker, Billerica, MA, USA) with chloroform-d (CDCl_3_) and methanol-d4 (CD_3_OD) using TMS as internal standard.

### 4.3. Ames Mutagenicity Assay

The *Salmonella typhimurium* mutagenicity assay was performed using the plate incorporation protocol with the strains TA100 and TA98 of *Salmonella typhimurium* [38]. Five different concentrations of the crude extract obtained from the leaves of *T. guianensis* were evaluated in this assay: 21.68, 16.26, 10.84, 5.42 and 2.71 mg/plate. The selection of the concentrations was based on the solubility limit of the samples in DMSO and on their toxicity in the TA98 and TA100 [38].

Toxicity was apparent both as a reduction in the number of his + revertants and as an alteration in the auxotrophic background (i.e., background lawn). The maximum volume of DMSO employed in the studies was 80 µL per plate.

The various concentrations of extract tested were added to 500 µL of buffer pH 7.4, 100 µL of bacterial culture and 2 mL of top agar. After agitation, the mixtures were poured on to plates containing minimum agar. The plates were incubated at 37 °C for 48 h and the his+ revertant colonies were manually counted. All experiments were performed in triplicate. The standard mutagens used as positive controls in experiments were 4-nitro-*o*-phenylenediamine (10 µg/plate) for TA98 and methylmethane sulfonate (260 µg/plate). DMSO (solvent) served as the negative control (80 µL/plate).

### 4.4. Preparation of Standard Solutions

Each sample was accurately weighed so that 0.1 g of each could be separately re-dissolved in 1 mL of DMSO. From these solutions, serial dilutions were made to obtain lower concentrations (5, 10, 25, 50, 75, 100, and 200 μg/mL) and then, were frozen at −20 °C, for later use.

### 4.5. Cell Lines and Cell Culture

The head and neck cell lines SCC14, SCC25, Fadu and HN13 (kindly provided by Rui Manuel Reis, Barretos Cancer Hospital) were used to determine the cytotoxic effect of different *T. guianensis* extract and fractions [39]. All cell lines were initially authenticated by short tandem repeat (STR) DNA typing according to the International Reference Standard for Authentication of Human Cell Lines using a panel of 8 (D5S818, D13S317, D7S820, D16S539, vWA, TH01, TPOX and CSF1P0) STR loci plus gender determination amelogenin (AMEL), using the fluorescent labeling primers as reported and tested for mycoplasma contamination [40]. The cell lines were maintained in Dulbecco’s modified Eagle’s medium (DMEM) (Sigma Aldrich, St. Louis, MO, USA), containing 10% fetal bovine serum (FBS) (Gibco-Life Technologies, Grand Island, NY, USA), 1% penicillin/streptomycin (Sigma Aldrich). Cells were incubated in a humidified atmosphere of 95% air and 5% CO_2_ at 37 °C.

### 4.6. Cell Viability Assay

The cell viability was assessed by MTT assay (Promega, Madison, WI, USA) as previous described [39,41]. To determine the IC_50_ values, 5 × 10^3^ cell lines/well were seeded in a 96 well plate and incubated at increased concentrations of different extract fractions of *T. guianensis*, under reduced FBS concentration (0.5%) for 72 h. The results were expressed as a percentage relatively to control cells (DMSO treatment). The IC_50_ values were calculated by nonlinear regression analysis using GraphPad Prism software (5.01 version, GraphPad Software, Inc., La Jolla, CA, USA). The growth inhibition (GI) was detected at fixed concentration of the 50 µg/mL, and cell lines were scored as highly sensitive (HS) with GI > 60%, moderately sensitive (MS) with GI between 40% and 60% and resistant with GI < 40% as previously described [39,42]. All the assays were done in triplicate and repeated at least three times.

### 4.7. Wound Healing Migration Assay

To assess the potential effect of *T. guianensis* fractions and crude extract in inhibition of cell migration, HN13 cell line was plated in 6 well plates (2.5 × 10^5^) in DMEM 10% FBS + 1% P/S and allowed to adhere overnight. After reaching 95% of confluence, mono-layer cells were washed with PBS, scraped with a plastic 200 μL pipette tip. The cell line was incubated with fixed concentrations of each fraction in DMEM 0.5% FBS + 1% P/S. Images of the wound were captured after 0 and 24 h by Olympus IX71 optical microscope (Olympus Optical CO. Hamburg, Germany) and the relative migration distance was measured by the following formula:

$$\mathrm{percentage}\;\mathrm{of}\;\mathrm{wound}\;\mathrm{closure}\left(\%\right)=\frac{100\times \left(A-B\right)}{A}$$

*A*, the width of cell wounds before incubation, and *B* the width of cell wounds after incubation [43].

### 4.8. Matrigel Invasion Assay

The invasion potential of *T. guianensis* fractions and crude extract was evaluated by BD BioCoat Matrigel invasion chambers Kit (BD Biosciences, San Jose, CA, USA), following manufacturer instructions and as previously described [44]. HN13 cell line (2.5 × 10^4^) cells were plated in the Matrigel-coated 24-well transwell inserts, containing DMEM (free-serum) and 2 µg/mL of the different compounds, as a chemo-attractant was used DMEM 10% FBS. Additionally, control inserts (without Matrigel), were used for measuring the migratory index of HN13 cell line exposed to different compounds.

HN13 cell line was allowed to invade and migrate for 24 h. Then, the insert membrane was fixed with methanol iced and stained with hematoxylin/eosin. The membranes were photographed at 40× magnification microscope and counted. The results were expressed in relation to the DMSO control (considered as 100% of invasion) as the mean percentage of invasion ± SD.

### 4.9. Western Blot

To assess apoptosis and MMP-2, HN13 cell line was cultivated in 6 well plates and after 90% plate confluence, cells were scraped in buffer lyses that contained 50 mM Tris (pH 7.6–8), 150 mM NaCl, 5 mM EDTA, 1 mM Na_3_VO_4_, 10 mM NaF, 10 mM Na_4_P_2_O_7_, 1% NP-40, and protease cocktail inhibitors. The cellular lysate was centrifuged at 13,000 rpm for 15 min in 5 °C and total protein of the supernatant was quantified by Bradford method. Briefly, 20 μg of proteins from lysates were resolved by 10% SDS-PAGE and transferred to nitrocelulose membranes in TransBlot Turbo transfer (Bio-Rad Laboratories, Hercules, CA, USA) and incubated in 5% nonfat dried milk in TBS-T for 1 h at room temperature before primary antibody overnight incubation with PARP total/cleaved (#9532, Cell Signaling Technology, Danvers, MA, USA), FAS (#4233, Cell Signaling Technology), CASPASE-3 (#14220, Cell Signaling Technology), CASPASE-9 (#9505, Cell Signaling Technology), TNFR1(#3736, Cell Signaling Technology), β-actin (#3700, Cell Signaling Technology) at 1:100 dilution, and at 1:500 dilution for MMP-2 (sc-58386-Santa Cruz Biotechnology, Dallas, TX, USA). After washing with TBS-T, membranes were incubated with the secondary antibody anti-rabbit (#7074, Cell Signaling Technology) at dilution 1:5000. Immune detection was done with ECL Western Blotting Detection Reagents in automatic ImageQuant mini LAS4000 (GE Healthcare Life Sciences, Pittsburgh, PA, USA). Densitometric data from Western blots were performed with ImageJ software (NIH-Scion Corporation, Bethesda, MD, USA). Caspase-3, FAS and TNFR1 values were normalized to β-actin levels and PARP cleaved to total PARP status. All experiments were carried out in triplicate.

### 4.10. Statistical Analysis

For Proliferation assay the results were expressed as the means ± SD. The results for Matrigel invasion assay were expressed in relation to the DMSO control (invaded cells) as the mean number of cells ± SD. Single comparisons between the different conditions studied were done using Student’s *t* test, and differences between groups were tested using two-way analysis of variance. Statistical analysis was done using GraphPad Prism version 5.01 (5.01 version, GraphPad Software, Inc.). The level of significance in all the statistical analyses was set at *p* ≤ 0.05. The results obtained were evaluated employing the statistical software Salanal and adopting the Bernstein et al. (1982) [45]. The data of Ames mutagenicity assay were assessed by analysis of variance (ANOVA) followed by a linear-regression. Furthermore, the mutagenic index (MI), which is the average number of revertants per plate divided by the average number of revertants per plate with the negative (solvent) control, was also calculated for each concentration. A sample was considered mutagenic when a dose–response relationship and a two-fold increase in the number of mutants (MI ≥ 2) with at least one concentration were observed.

## 5. Conclusions

The present study showed cytotoxic activity of *T. guianensis* extract on oral cancer cells lines, as well as an ability to inhibit tumor migration and invasion, constituting a putative anticancer agent, alone or in combination with classic chemotherapy and radiotherapy approaches. Nevertheless, further studies are needed to identify the mechanisms by which *T. guianensis* extract acts as inhibitors of cell proliferation.

## Figures and Tables

**Table 1:** Revertant/plate, standard deviation and mutagenicity index (MI) in the strains TA98 and TA100 of *S. typhimurium* after treatment with different doses of the extract from *T. guianensis*, without (−S9) and with (+S9) metabolic activation.

Treatment	TA98				TA100			
	−S9		+S9		−S9		+S9	
	Mean ± SD	MI	Mean ± SD	MI	Mean ± SD	MI	Mean ± SD	MI
NC	16.7 ± 6.0	–	41.7 ± 5.69	–	121.7 ± 18.2	–	97.00 ± 40.85	–
Extract (µg/mL)	–	–	–	–	–	–	–	–
4.25	56.0 ± 11.0 *	3.3	140.3 ± 87.5 **	3.4	130.0 ± 37.2	1.1	198.7 ± 24.5 *	2.0
8.67	67.3 ± 16.3 *	4.0	180.7 ± 42.2 *	4.3	118.0 ± 74.6	1.0	232.0 ± 8.9 *	2.4
16.68	136.0 ± 16.7 *	8.1	309.7 ± 74.6 *	7.4	68.7 ± 33.1	0.6	250.7 ± 35.2 *	2.6
24.09	186.0 ± 29.9 *	11.1	423.7 ± 60.5 *	10.2	110.7 ± 11.5	0.9	306.3 ± 31.2 *	3.2
30.97	246.3 ± 43.7 *	14.7	315.0 ± 10.5 *	7.6	119.7 ± 29.0	1.0	308.3 ± 41.4 *	3.2
PC	426.3 ± 40.3 *	–	2839.7 ± 515.3 *	–	471.0 ± 194.2 *	–	1257.3 ± 159.3 *	–

Mean ± SD: Revertants Mean Frequency per plate ± Standard Deviation; MI: Mutagenicity Index; NC: Negative Control (DMSO − 80 µL/plate); PC: Positive Control 4-nitro-o-phenylenediamine (10 μg/plate − TA98 (−S9)), Methylmethane sulfonate (260 μg/plate − TA100 (−S9)) or 2-Aminoanthracene (5 μg/plate − TA98 (+S9) and TA100 (+S9)); * *p* < 0.05 and MI > 2; ** MI > 2.

**Table 2:** Classification and IC_50_ value response to different fractions of the *T. guianensis* extract in HNSCC cell lines.

Cell Line	Anatomic Site	IC_50_ Value (Mean ± DP) mg/mL			
–	–	C1	C2	C3	C4
SCC14	Hypopharynx	0.029 ± 0.0007	0.014 ± 0.002	0.058 ± 0.002	0.074 ± 0.002
SCC25	Oral Cavity	0.244 ± 0.048	0.240 ± 0.026	0.349 ± 0.056	0.592 ± 0.020
Fadu	Hypopharynx	0.050 ± 0.002	0.023 ± 0.002	0.056 ± 0.003	0.186 ± 0.018
HN13	Tongue	0.028 ± 0.0002	0.015 ± 0.002	0.045 ± 0.004	0.058 ± 0.001

**Table 3:** Growth inhibition to different fractions of the *T. guianensis* extract in HSCC cell lines.

Cell Line	GI Classification at 0.05 mg/mL (%)			
–	C1	C2	C3	C4
SCC14	MS (58.3%)	HS (63.3%)	MS (55.3%)	MS (46.7%)
SCC25	R (25.3%)	R (28%)	R (25%)	R (0%)
Fadu	MS (47%)	HS (66.3%)	MS (50.3%)	MS (47.3%)
HN13	MS (58.3%)	HS (66.3%)	MS (55.3%)	MS (46.6%)

HS: highly sensitive; MS: moderate sensitive and R: resistant.

## Supplementary Materials

Supplementary materials can be found at www.mdpi.com/1422-0067/17/11/1839/s1.
