# Supplementary material for: Tapirira guianensis Aubl. Extracts Inhibit Proliferation and Migration of Oral Cancer Cells Lines
Source: Int J Mol Sci. 2016 Nov 8;17(11):1839. doi: 10.3390/ijms17111839 (PMC5133839; doi:10.3390/ijms17111839)
Supplement: Supplementary file 1 [file ijms-17-01839-s001.pdf]

Supplementary Materials: *Tapirira guianensis* Aubl. Extracts Inhibit Proliferation and Migration of Oral Cancer Cells Lines

Renato José Silva-Oliveira, Gabriela Francine Lopes, Luiz Fernando Camargos, Ana Maciel Ribeiro, Fábio Vieira dos Santos, Richele Priscila Severino, Vanessa Gisele Pasqualotto Severino, Ana Paula Terezan, Ralph Gruppi Thomé, Hélio Batista dos Santos, Rui Manuel Reis and Rosy Iara Maciel de Azambuja Ribeiro

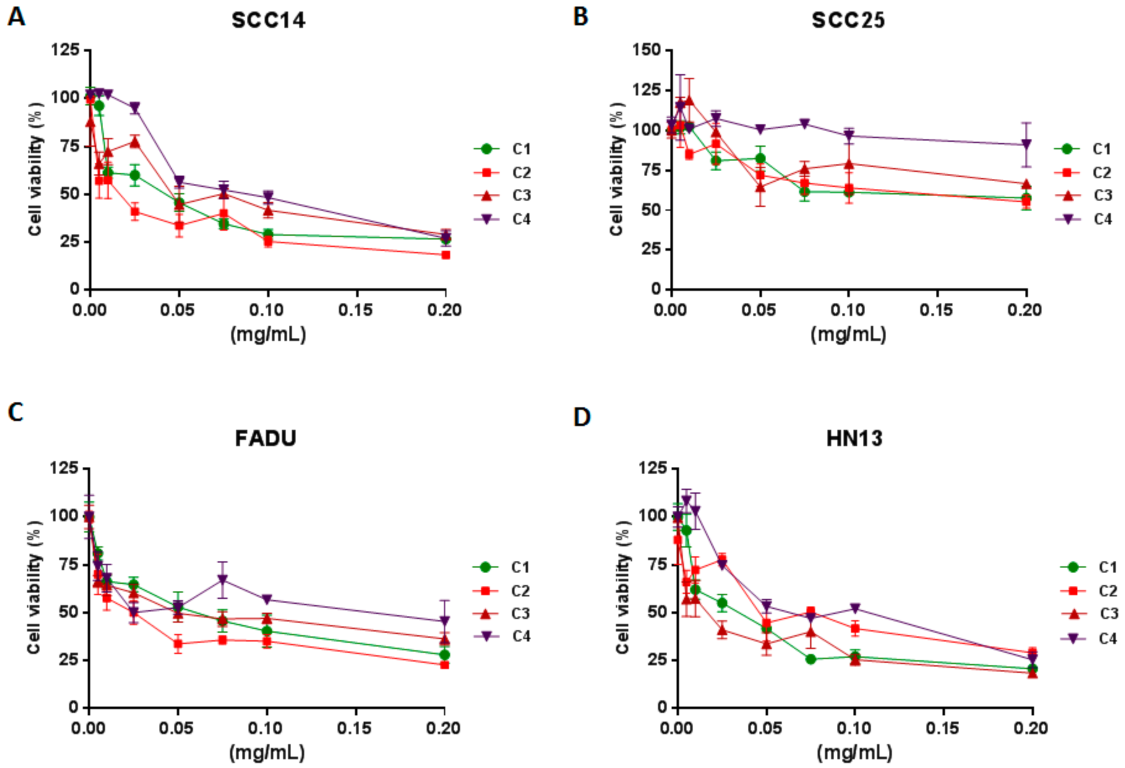

Figure S1. A representation of the proliferation and survival curves of the head and neck tumor cell lines (SCC14 (**A**), SCC25 (**B**), FADU (**C**), HN13 (**D**)).
